# Kernel-based variance component estimation and whole-genome prediction of pre-corrected phenotypes and progeny tests for dairy cow health traits

**DOI:** 10.3389/fgene.2014.00056

**Published:** 2014-03-24

**Authors:** Gota Morota, Prashanth Boddhireddy, Natascha Vukasinovic, Daniel Gianola, Sue DeNise

**Affiliations:** ^1^Department of Animal Sciences, University of Wisconsin-MadisonMadison, WI, USA; ^2^Zoetis Inc.Kalamazoo, MI, USA; ^3^Department of Biostatistics and Medical Informatics, University of Wisconsin-MadisonMadison, WI, USA; ^4^Department of Dairy Science, University of Wisconsin-MadisonMadison, WI, USA

**Keywords:** dairy cow, genetic variance, kernel method, non-additive effect, whole-genome prediction

## Abstract

Prediction of complex trait phenotypes in the presence of unknown gene action is an ongoing challenge in animals, plants, and humans. Development of flexible predictive models that perform well irrespective of genetic and environmental architectures is desirable. Methods that can address non-additive variation in a non-explicit manner are gaining attention for this purpose and, in particular, semi-parametric kernel-based methods have been applied to diverse datasets, mostly providing encouraging results. On the other hand, the gains obtained from these methods have been smaller when smoothed values such as estimated breeding value (EBV) have been used as response variables. However, less emphasis has been placed on the choice of phenotypes to be used in kernel-based whole-genome prediction. This study aimed to evaluate differences between semi-parametric and parametric approaches using two types of response variables and molecular markers as inputs. Pre-corrected phenotypes (PCP) and EBV obtained for dairy cow health traits were used for this comparison. We observed that non-additive genetic variances were major contributors to total genetic variances in PCP, whereas additivity was the largest contributor to variability of EBV, as expected. Within the kernels evaluated, non-parametric methods yielded slightly better predictive performance across traits relative to their additive counterparts regardless of the type of response variable used. This reinforces the view that non-parametric kernels aiming to capture non-linear relationships between a panel of SNPs and phenotypes are appealing for complex trait prediction. However, like past studies, the gain in predictive correlation was not large for either PCP or EBV. We conclude that capturing non-additive genetic variation, especially epistatic variation, in a cross-validation framework remains a significant challenge even when it is important, as seems to be the case for health traits in dairy cows.

## Introduction

In animal breeding, a main goal is to attain genetic gain for economically important traits in subsequent generations. In the genomic era, dense molecular genetic markers disseminated across the entire genome can be combined with extant information, such as pedigrees, to obtain more accurate predictions of the genetic values of candidate animals and to make selection decisions. Whole-genome prediction methods that incorporate all available DNA marker information have been proposed for this purpose (Meuwissen et al., 2001; Gianola et al., 2003), and these are now extensively used in animal breeding (e.g., de los Campos et al., 2013a) and deemed as a promising tool in plant breeding (e.g., Nakaya and Isobe, 2012), preventive medicine, and clinical decision making (e.g., de los Campos et al., 2010a; Vazquez et al., 2012).

The main rationale behind whole-genome approaches is to capture signal via markers irrespective of the statistical significance of individual markers. There is increasing evidence that complex traits are the product of synergistic forces spanned by large numbers of genetic polymorphisms within the genome (e.g., Huang et al., 2012). This reaffirms the view that genetic interaction is important and that genotypes and phenotypes may be linked in a non-linear manner that may not be amenable to parametric modeling. The issue is particularly pertinent to the animal and plant breeding domains, which have been dealing with complex trait genetics scientifically since the beginning of the 20th century (Fisher, 1918; Wright, 2010). While breeding exploits additive inheritance, developing flexible phenotypic prediction machines that perform well regardless of the underlying genetic makeup is desirable. Further, accomodating non-additive effects in a model may enhance predictive ability of breeding values.

Gianola et al. (2006); Gianola and van Kaam (2008); Gianola et al. (2010) have laid groundwork for semi-parametric whole-genome regression methods that address non-additive variation, albeit in a non-explicit manner. Bayesian kernel ridge regression [a form of reproducing kernel Hilbert spaces (RKHS) regression] and Bayesian neural networks are two major smoothers that have been used to date. The semi-parametric methods have resulted in a somewhat greater predictive ability than that delivered by linear additive smoothers in real data, including Jersey cows (Gianola et al., 2011), heterogeneous mice (Okut et al., 2011), broiler chickens (González-Recio et al., 2008, 2009; Long et al., 2010), and wheat (Long et al., 2011a; Pérez-Rodríguez et al., 2012). In RKHS, typically a Gaussian kernel is employed as a basis function, to estimate conditional expectations. Its essence is to condense hundreds of thousands of genetic markers into a positive (semi) definite kernel matrix of order *n* × *n* (*n* is the number of phenotypes) by creating genetic relatedness in terms of “spatial” distance on a certain metric space. Although pedigree and genomic relationship matrices, **A** and **G**, respectively, are valid kernels in RKHS, further smoothing of the relatedness conveyed by **A** and **G** may enable better prediction under complex gene action.

On the other hand, the gain obtained from semi-parametric methods has been smaller when predicted transmitting ability (PTA) or estimated breeding value (EBV) was used as the response variable (Long et al., 2011a; Morota et al., 2013). Thus, further research is needed to fully exploit the theoretical advantage of semi-parametric whole-genome regression. The choice of target phenotypes has been discussed in conventional genetic evaluation schemes (VanRaden and Wiggans, 1991), genome-enabled selection (Garrick et al., 2009; Guo et al., 2010; Ostersen et al., 2011; Boddhireddy et al., 2014) and quantitative trait loci (QTL) mapping (Thomsen et al., 2001), but mostly in the context of additive genetic effects. This is particularly relevant to dairy cattle breeding for milk where bulls do not posses milk records and heavy use is made of artificial insemination and progeny tests. For example, the EBV of a bull is a smoothed weighted average of records from all available relatives assuming additive inheritance (the transmission rule is encoded in matrix **A**) and a daughter yield deviation (DYD) is the average of a bull's daughter performance adjusted for systematic effects, as well as for genetic effects of the daughter's dams. A de-regressed proof (DRP) is similar to DYD and can be derived from EBV; it removes parent average effects and eliminates shrinkage inherent to EBV.

It is conceivable that the type of response variables used to regress on kernels influences predictive performance. For instance, EBV mainly encodes additive genetic effects and depends on the narrow sense heritability of a target trait. Variation in EBV, especially if it has a high reliability, is expected to reflect mostly additive genetic components, whereas pre-corrected phenotypes (PCP) may be affected by other sources of variation, both environmental and genetic.

One concern is that the pre-processing of phenotypes by fitting linear mixed models prior to conducting a genome-enabled prediction may break underlying genotype-phenotype maps. It may be that kernel methods are relatively better than their parametric counterparts when applied to PCP than when used with EBV as response variable. After all, predicting an average (such as EBV) is easier than predicting a phenotype, so one might expect larger differences among prediction machines when applied to PCP. The objective of this study was to quantify the type and amount of genetic variance in complex traits and to investigate differences between predictive performance of non-parametric and parametric kernels when applied to two types of response variables: PCP and EBV, both derived from raw phenotypes.

## Materials and methods

### Data

The full dataset included 4482 dairy cows genotyped with 54,609 whole-genome SNPs on the Illumina BovineSNP50 BeadChip. EBV and PCP were available for six health traits: ketosis (KET), displaced abomasum (DA), retained placenta (RP), lameness (LAME), metritis (METR), and clinical mastitis (CM). We chose EBV because a recent study (Boddhireddy et al., 2014) found that predictive correlations obtained using EBV were consistently greater than those obtained using deregressed EBV. The same study demonstrated that the predictive correlations dropped even more when accuracies of estimated EBVs are low, which was the case for the health traits we analyzed in this paper. Further, Guo et al. (2010) reported that deregressed EBV yielded slightly lower reliabilities on simulated data. PCP was obtained by fitting a least squares model to raw binary phenotypes (presence or absence) using parity, herd, year, and season effects as explanatory variables. Similarly, EBV was predicted via best linear unbiased prediction (BLUP) using an **A** matrix considering 14,685 animals, dating back 10 generations on average. The number of animals with both genotypes and phenotypes available varied across traits. All animals had EBV for every trait, while only 2886, 4227, and 3622 animals with PCP were available for KET, DA, and RP, respectively. Average values of reliabilities associated for these EBV were 0.21, 0.35, 0.24, 0.28, 0.49, and 0.23 for KET, DA, RP, LAME, METR, and CM, respectively. Monomorphic markers were not considered and SNPs that had a minor allele frequency (MAF) less than 0.05, resulting in 41,266 markers used for the analysis. Missing genotypes were replaced locus by locus by sampling alleles from a Bernoulli distribution with the marginal allele frequency used as the parameter. A study in pine has shown that predictions are stable with respect to various imputation methods (Zapata-Valenzuela et al., 2013).

### Choice of kernels

We aimed to capture signal from genotypes to phenotypes through construction of a kernel matrix **K**. Three non-parametric and three parametric kernels were considered. The non-parametric Gaussian kernel (**GK**) can be constructed by embedding a vector of SNPs into the Euclidean metric space. The spatial genetic distance between two individuals with corresponding vectors of genotypes **x**_*i*_ and **x**_*j*_ is given by the squared Euclidean norm *k*(**x**_*i*_, **x**_*j*_) = exp(−θ||**x**_*i*_ − **x**_*j*_||^2^), where a positive bandwidth parameter θ controls overall smoothness of the function. This kernel is known to approximate a diffusion kernel well, with the latter defined on a discrete non-Euclidean metric space (Morota et al., 2013). We built two types of Gaussian kernels that differed with respect to allele coding (Long et al., 2011b). An additive Gaussian kernel, hereinafter denoted as **GK**_A_, was based on coding marker genotypes in an additive manner, such as “aa” → 0, “Aa” → 1, “AA” → 2. Similarly, coding genotypes “aa”, “Aa” and “AA” as -0.5, 0.5, and -0.5, respectively, leads to a dominance Gaussian kernel (**GK**_D_). The third non-parametric kernel, aimed to capture additive by dominance epistasis, was constructed by taking the Hadamard (element by element) product of matrices, that is, **GK**_A_#**GK**_D_, following Henderson (1985). This parameterization assumes no linkage and linkage equilibrium (LE). With respect to the parametric kernels, the first approach was an additive genomic relationship matrix **G** (VanRaden, 2008) based on the additive genotype matrix (**X**_A_). Subsequently, its dominance counterpart **D** was derived by constructing a dominance contrast between marker genotypes (**X**_D_). Under dominance and Hardy-Weinberg equilibrium, the expectation and variance of genotypes at a locus (*x*_*i*_) are given by 2*p*_*i*_(1 − *p*_*i*_) and 2*p*_*i*_(1 − *p*_*i*_)[1 − 2*p*_*i*_(1 − *p*_*i*_)], respectively, where *p*_*i*_ is the allele frequency of a reference allele (Su et al., 2012). Using the same logic as above, the parametric version of an additive by dominance epistasis kernel is given by **G**#**D**. The first three kernels (**GK**_A_, **GK**_D_, and **GK**_A_#**GK**_D_) incorporate markers into the regression equation non-parametrically in a non-linear manner, whereas the other three kernels possess a parametric interpretation and are linear on additive or dominance relationships.

### Bayesian kernel ridge regression

The procedure is as in Morota et al. (2013). A standard quantitative genetics model attempts to separate observed values (**y**) into genetic (**g**) and residual (ϵ) components by setting up an equation **y** = **g** + ϵ. The residual term ϵ may contain model misspecification and environmental effects not considered in the analysis. The genetic signal is regarded as an unknown conditional expectation function taking the form **g** = **K**α under the representer theorem (e.g., de los Campos et al., 2010b). Here, **K** is one of the kernels discussed above and the coefficient α is the solution that optimizes ℓ(α|λ) = (**y** − **K**α)′(**y** − **K**α) + λα′**K**α. This is equivalent to fitting **y** = **K**α + ϵ, with α and ϵ following independent *N*(0, **K**^−1^σ^2^_α_) and *N*(0, **I**σ^2^_ϵ_) distributions, respectively; λ is the ratio of variance components, σ^2^_ϵ_/σ^2^_α_. Thus, within the framework of this particular RKHS regression model, the response and the kernel are linked in a linear fashion, while the SNP covariates enter either linearly or non-linearly into the kernels. The prediction of genetic values is given by the estimated conditional expectation function $\hat{g}=K\hat{\alpha }$.

All unknown terms, including the variance components, can be inferred from a posterior distribution using Gibbs sampling. Scaled inverse chi-squared distributions with degrees of freedom equal to 5 and a scale parameter proportional to the phenotypic variance times 0.5 were used as priors for the two variance parameters. We employed a Markov chain of 50,000 iterations, with the first 20,000 discarded as burn-in. Thinning rate was 10, yielding 3000 samples for posterior inference of each parameter of interest. For EBV, reliabilities associated with those EBV were used as weights.

### Weights of kernels

The contribution of each kernel was evaluated through “kernel averaging” (i.e., multiple kernel learning) as proposed in de los Campos et al. (2010b). The three parametric kernels **G**, **D**, and **G**#**D** were used to quantify the amount of variance that can be attributed to marked additive, dominance, and additive by dominance epistasis as in standard variance component estimation. Here, the “average” kernel **K** takes the form $K=G\frac{\sigma_{\text{G}}^{2}}{{\tilde{\sigma }}_{\text{K}}^{2}}+D\frac{\sigma_{\text{D}}^{2}}{{\tilde{\sigma }}_{\text{K}}^{2}}+(G\#D)\frac{\sigma_{\text{GD}}^{2}}{{\tilde{\sigma }}_{\text{K}}^{2}}$, where σ^2^_G_, σ^2^_D_, σ^2^_GD_ are variance components linked to kernels **G**, **D**, and **GD**, respectively, and $\tilde{\sigma }$^2^_K_ = σ^2^_G_ + σ^2^_D_ + σ^2^_GD_. Thus, σ^2^_G_/$\tilde{\sigma }$^2^_K_, σ^2^_D_/$\tilde{\sigma }$^2^_K_, σ^2^_GD_/$\tilde{\sigma }$^2^_K_ can be viewed as the relative contributions of the kernels to the marked genetic variation in the population. The larger the corresponding weights are, the larger the contribution of a specific type of genetic variance to overall variation. We also quantified weights by fitting only additive and dominance kernels, to evaluate potential bias due to model misspecification.

The bandwidth parameter (θ) attached to a Gaussian kernel can be either inferred within a Bayesian MCMC sampling framework or evaluated over a grid of values of θ. The approach adopted here was to use the kernel averaging described earlier in an MCMC context. Two non-parametric kernels of the same type were created using “extreme” values of bandwidth parameters so that the means of the average off-diagonal elements of the corresponding kernels were 0.12 and 0.90, respectively. Thus, any of the three kernels created was based on both local (0.12) and global (0.90) similarities among individuals. Parametric kernels do not involve this bandwidth parameter.

The RKHS regression models were fitted using functions obtained from the R package BGLR (bglr.r-forge.r-project.org).

### Assessment of predictive ability

The predictive ability of the models was assessed using a 10-fold cross-validation (CV) by splitting the data randomly into 10 disjoint sets of about equal size. Nine sets were used as training data to predict masked phenotypes of animals in the remaining set (testing). Predictive performance was measured as Pearson's correlation between predicted and observed values in the testing set. To smooth variability of the CV distribution, the average of five 10-fold CV was calculated. The predictive performance of three combinations of non-parametric kernels (**GK**_A_, **GK**_D_, and **GK**_A_ + **GK**_D_ + **GK**_A_#**GK**_D_) and one combination of parametric kernels (**G** + **D** + **G**#**D**) was compared against the benchmark kernel **G**, which is equivalent to genomic best linear unbiased prediction.

## Results

**Figure 1:**
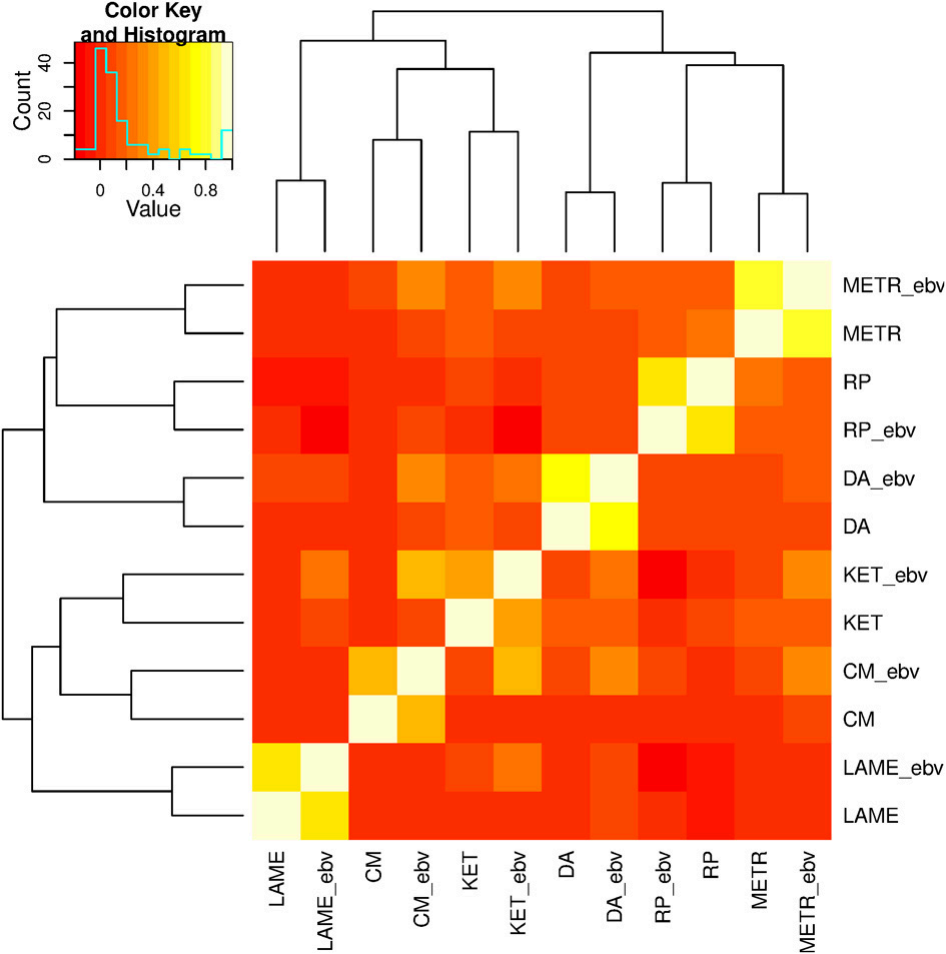
**Correlations among six health traits: ketosis (KET), displaced abomasum (DA), retained placenta (RP), lameness (LAME), metritis (METR), and clinical mastitis (CM)**. Variable names followed by "_ebv" denote estimated breeding values (EBV).

Pair-wise correlations among the 12 response variables are displayed as a heatmap in Figure 1. Correlations among the 6 PCP, the 6 EBV, and the 6 pairs of PCP and EBV on the same traits ranged between −0.03 and 0.21, −0.19 and 0.51, and 0.41 and 0.78, respectively. A hierarchical clustering, joined the two response variables of the same trait as expected, and six traits were clustered into two large groups: (1) METR, RP, and DA, and (2) LAME, CM, and KET.

A summary of the estimated variance components is shown in Table 1. Here, *V*_*G*_, *V*_*D*_, *V*_*GD*_, and *V*_*K*_ represent marked additive (σ^2^_*G*_), marked dominance (σ^2^_*D*_), marked additive by dominance (σ^2^_*GD*_), and total marked genetic variance ($\tilde{\sigma }$^2^_*K*_ = σ^2^_*G*_ + σ^2^_*D*_ + σ^2^_*GD*_), respectively, and *H*^2^ is estimated broad sense heritability. Narrow sense heritability estimates of PCP (*V*_*G*_/*V*_*P*_) ranged from 0.05 (RP) to 0.09 for KET. This is consistent with literature reports for health traits (e.g., Heringstad et al., 2005; Heringstad, 2010; Koeck et al., 2012). We observed that non-additive genetic variances were major sources of genetic variation in PCP, whereas additivity had the largest contribution to variability of EBV as one would expect. Additive by dominance epistasis followed by dominance had the largest contribution to variation in health PCP, which is in agreement with pedigree-based analyses (e.g., Hoeschele, 1991; Palucci et al., 2007) suggesting the hypothesis that non-additive genetic variances are important for fitness related traits (e.g., fertility). For all PCP traits, the amount of non-additive genetic variance was greater than the additive variance. Unexpectedly, a sizeable amount of epistatic variances was also captured for EBV of DA, LAME, METR, and CM, which is believed to be embedding solely additive variability. On the other hand, the contribution of epistasis for EBV of KET and RP was negligible. A reason is that the interpretation of variances from marker-based models should not be the same as that of variance estimates from pedigree data. Also, these EBV were for health traits having low narrow sense heritability and, hence, low reliability. A third reason is that the variance partition applies to averages, producing a much larger contribution of genetic variances than when the partition is for single records. Estimates of broad sense heritability ranged from 0.33 to 0.52 for PCP and 0.29 to 0.78 for EBV. As mentioned above, the variance among EBV was small, because these are averages. The phenotypic variances among EBV for the six traits ranged between 0.0057 and 0.0157. Hence, this magnifies the contribution of genetic variances compared to decompositions obtained with PCP.

**Table 1:** **Estimated ratios of variance components (weights) for ketosis (KET), displaced abomasum (DA), retained placenta (RP), lameness (LAME), metritis (METR), and clinical mastitis (CM) using parametric multiple-kernel learning**.

**Traits**	**Types**	**Variance components**						
		***V*_*G*_/*V*_*P*_**	***V*_*D*_/*V*_*P*_**	***V*_*GD*_/*V*_*P*_**	***H*^2^**	***V*_*G*_/*V*_*K*_**	***V*_*D*_/*V*_*K*_**	***V*_*GD*_/*V*_*K*_**
KET	PCP	0.09 (0.10)	0.13 (0.14)	0.14	0.35 (0.24)	0.24	0.36	0.40
	EBV	0.25 (0.24)	0.03 (0.04)	0.01	0.29 (0.28)	0.84	0.12	0.04
DA	PCP	0.06 (0.08)	0.09 (0.10)	0.25	0.40 (0.18)	0.16	0.22	0.62
	EBV	0.39 (0.30)	0.04 (0.05)	0.30	0.73 (0.36)	0.53	0.05	0.41
RP	PCP	0.05 (0.07)	0.09 (0.11)	0.35	0.50 (0.18)	0.11	0.18	0.71
	EBV	0.27 (0.23)	0.03 (0.03)	0.07	0.37 (0.26)	0.73	0.07	0.20
LAME	PCP	0.06 (0.07)	0.07 (0.09)	0.39	0.52 (0.16)	0.12	0.14	0.75
	EBV	0.39 (0.30)	0.03 (0.08)	0.27	0.70 (0.38)	0.56	0.05	0.39
METR	PCP	0.06 (0.07)	0.07 (0.08)	0.21	0.33 (0.15)	0.17	0.21	0.62
	EBV	0.31 (0.26)	0.05 (0.07)	0.42	0.78 (0.34)	0.39	0.07	0.54
CM	PCP	0.06 (0.07)	0.07 (0.09)	0.26	0.39 (0.16)	0.15	0.18	0.66
	EBV	0.36 (0.29)	0.02 (0.05)	0.16	0.54 (0.34)	0.66	0.04	0.29

*The epistatic kernel was created from the Hadamard product of additive and dominance kernels. Pre-corrected phenotype (PCP) and estimated breeding value (EBV) were used as phenotypes. V_G_, V_D_, V_GD_, and V_K_ represent marked additive (σ^2^_G_), marked dominance (σ^2^_D_), marked additive by dominance (σ^2^_GD_), and total marked genetic variance ($\tilde{\sigma }$^2^_K_ = σ^2^_G_ + σ^2^_D_ + σ^2^_GD_), respectively, H^2^ is estimated marked broad sense heritability. Values in parentheses are estimated weights when kernels were fitted separately*.

**Figure 2:**
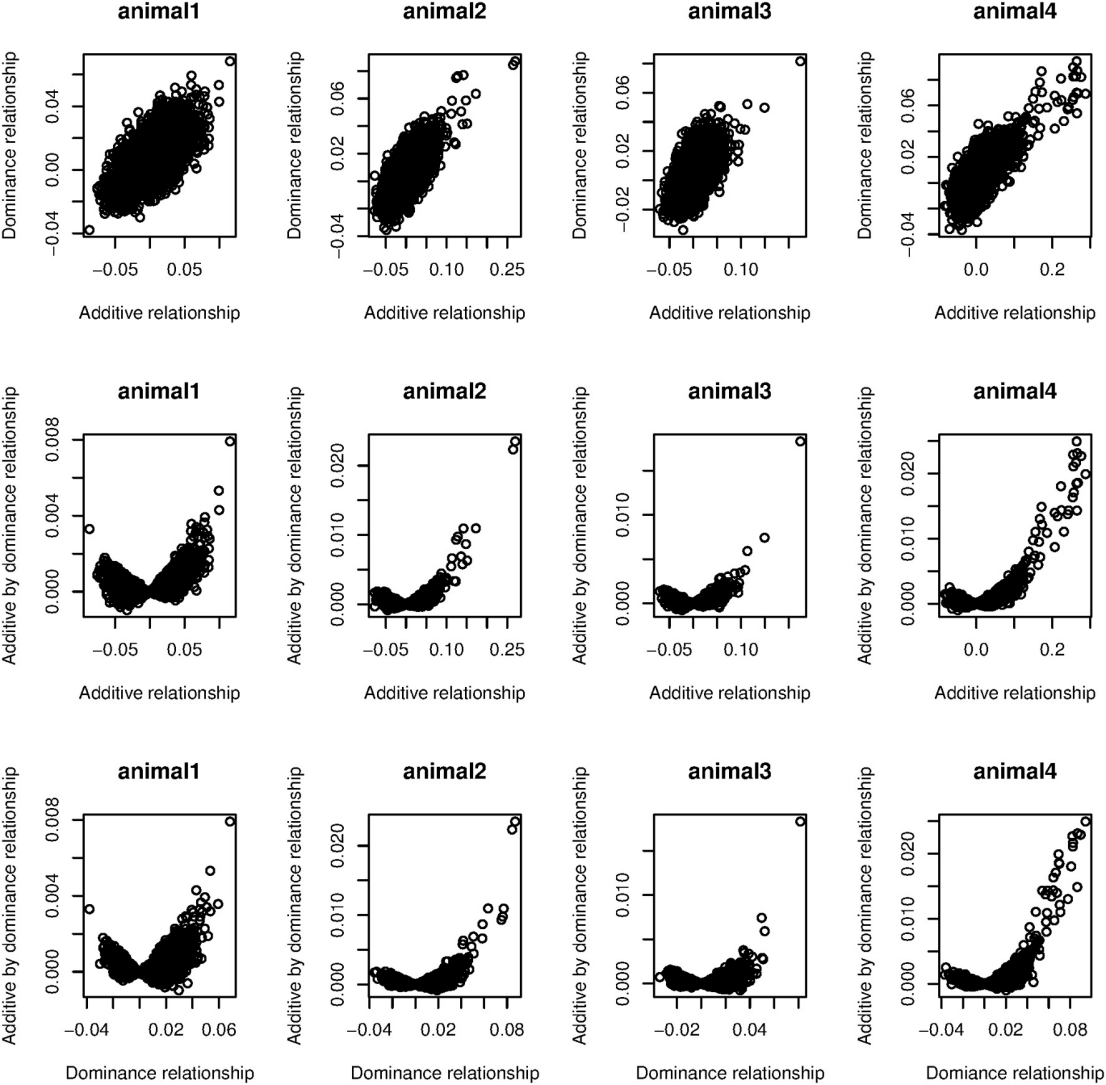
**Scatter plots of relationships among additive (**G**), dominance (**D**), and additive by dominance (**GD**) taken from four randomly sampled animals**.

Values in parentheses in Table 1 are the estimated weights (contribution to total variance) when only additive and dominance kernels were fitted. All dominance kernel weights so estimated were slightly higher or equal for both PCP and EBV than under the full model. On the other hand, estimated weights in the two kernels model for PCP were higher, whereas for EBV, the opposite trend was observed. By comparing the full with reduced models, we noted that most epistatic signal come from the residual variances. This suggests that the models were not able to separate additive by dominance from residual variances, probably because most off-diagonal elements in **G**#**D** are zero, which is close to an identity matrix. In our data set, averages of absolute values of off-diagonals were 0.02, 0.01, and 0.0003 for **G**, **D**, and **GD**, respectively. Figure 2 depicts scatter plots of relationships among **G**, **D**, and **GD** that are taken from four randomly sampled animals. We see that the vast majority of off-diagonal components of **GD** are concentrated around zero. One approach to mitigate this problem is to use more strongly related animals, so that off-diagonals of **G**#**D** would be further away from zero.

**Figure 3:**
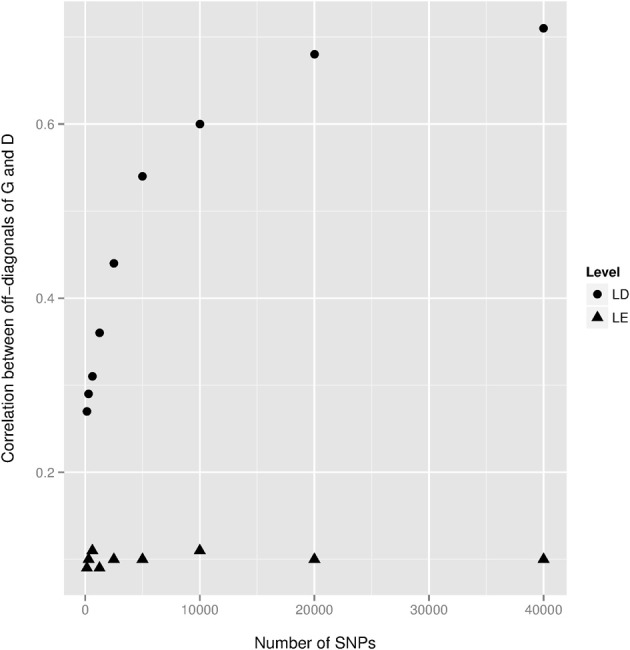
**Correlations between off-diagonal elements of the additive genomic relationship matrix **G** and of the dominance relationship matrix **D** as a function of the number of SNPs**. Genotypes were both randomly sampled from the present study (Level = LD) and via a computer simulation locus by locus (Level = LE) with an average minor allele frequency equal to 0.35. The averages of the *r*^2^ linkage disequilibrium (LD) statistic between adjacent markers were 0.18 and 0.008 for the real and simulated datasets, respectively.

We observed a small dominance contribution for EBV and found that the correlation between corresponding elements of **G** and **D** was 0.70. This dependency is also highlighted in the first row of Figure 2. We found that when a larger number of SNPs was used to construct **D**, the off-diagonal elements of this kernel became more strongly correlated with those of **G**. This is illustrated in Figure 3, where additive and dominance kernels were created from randomly sampled genotypes from this study, where average adjacent linkage disequilibrium (LD) was 0.18 when using the *r*^2^ metric. Genotypes under a LE scenario were created via computer simulation with an average MAF of 0.35. The number of animals was fixed as in this study (*n* = 4,482), while varying the number of markers from 150 to 40,000. Under LD, the two kernels became more similar as the number of SNP increase, suggesting that a partition of marked variance into additive and dominance components may be difficult to attain, producing misleading results unless the kernel takes somehow into account the ratio *p*/*n* when the number of markers is much larger than the number of animals, *p* >> *n*. On the other hand, correlations between off-diagonal elements of the additive and dominance relationship matrices remained constant at small values when LD was absent.

**Figure 4:**
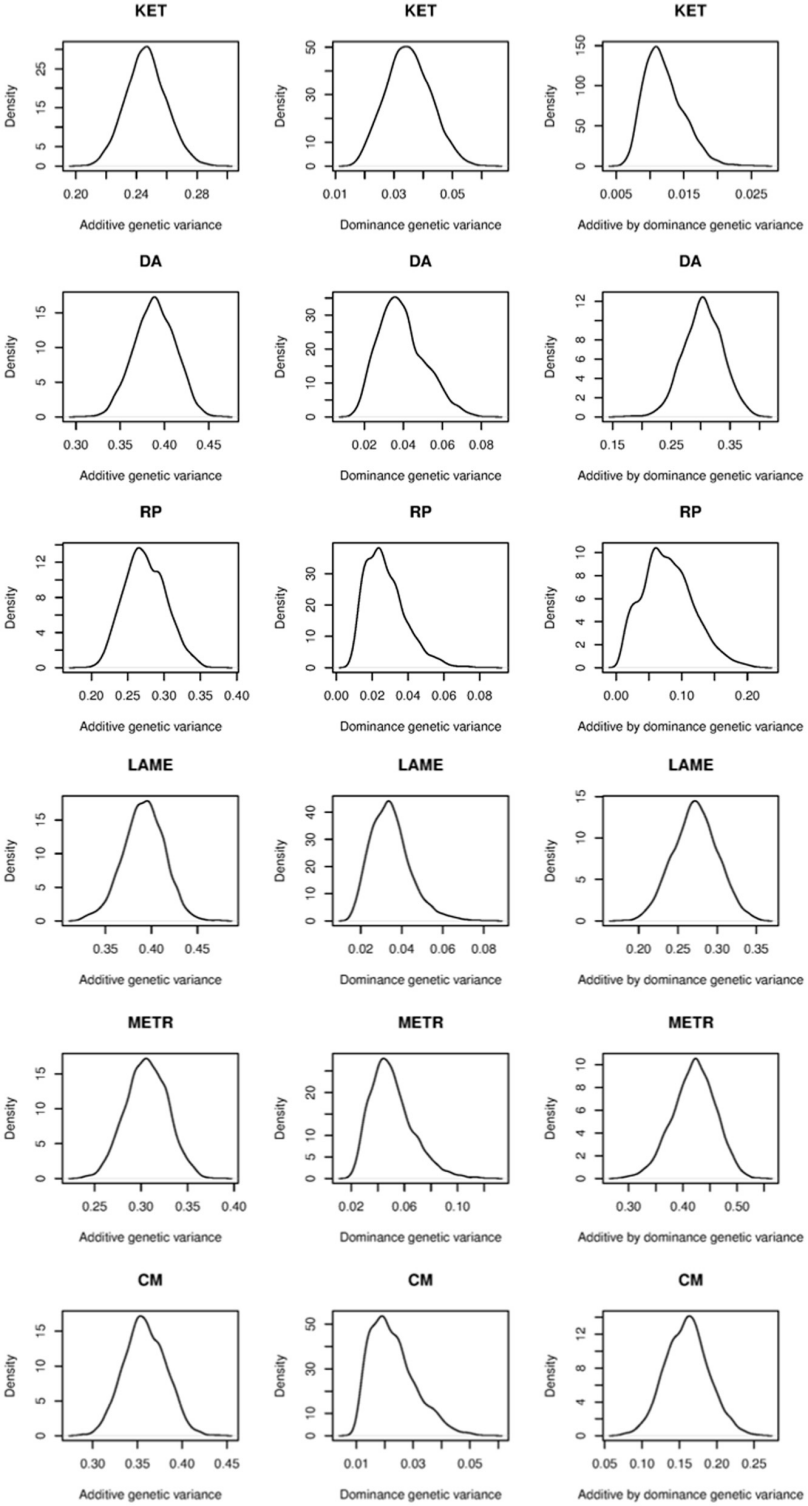
**Posterior density plots of the ratios of variance components for ketosis (KET), displaced abomasum (DA), retained placenta (RP), lameness (LAME), metritis (METR), and clinical mastitis (CM)**. Estimated breeding value was used as phenotype.

Table 2 shows predictive correlations for the kernels employed. The non-parametric kernels **GK**_A_ and **GK**_ALL_ yielded slightly better predictive performance than the additive genomic-BLUP (**G**) for all traits regardless of the type of response variable used. Fitting the three parametric kernels (**G**, **D**, and **G**#**D**) together gave a slightly better predictive performance than **G** alone in most cases. Overall, either the Gaussian additive kernel alone, or the three non-parametric kernels (Gaussian additive, dominance, and additive by dominance) fitted jointly delivered the best performance. The Gaussian kernel derived from the dominance contrasts did not perform well unless the Gaussian additive kernel was fitted together. These results indicate presence of marked non-additive genetic variation in PCP, and that kernels that make use of non-additive sources of information may deliver better predictions. Non-parametric kernels performed better than parametric counterparts for EBV. However, the gain in predictive ability from the use of non-parametric kernels was similar for PCP and EBV, at least as measured by correlation. This gain was marginal and varied between 0.01 and 0.03 over traits, indicating that the non-parametric kernels were unable to exploit presence of non-additive genetic variation for PCP effectively, at least for these traits. Our observation is congruent with a recent study in pigs, where additive and non-additive genetic variances were obtained and prediction was made using parametric kernels (Su et al., 2012). Although these authors reported large non-additive genetic variances, use of genomic BLUP accommodating additive, dominance, and additive by additive epistasis yielded a marginal gain compared to the additive genomic relationship kernel alone. As stated previously, additive and dominance kernels are correlated by construction and these two kernels were also strongly correlated with additive by dominance kernels. These observations indicate that the LE assumption of Kempthorne (1954) is violated and suggest that use of a single Gaussian kernel aimed to capture total genetic variation may be preferred for prediction purposes compared to parameterizing into three genetic components. Perhaps the variance component estimates reported in Su et al. (2012) and the values obtained in our study are unstable or are biased upwards because of lack of orthogonality among parametric kernels and, if this is the case, a significant gain would not be achieved with prediction models aiming to capture non-additive genetic variation using naively structured kernels. The possibility of having unstable estimates may be excluded for our case as the posterior density of the ratios of variance components for EBV were unimodal (Figure 4). Genomic relationship kernels that are “orthogonal” to each other could enhance prediction ability but such kernels are not straightforward to construct.

**Table 2:** **Predictive correlation for ketosis (KET), displaced abomasum (DA), retained placenta (RP), lameness (LAME), metritis (METR), and clinical mastitis (CM) using various kernels and the average of five 10-fold cross-validation**.

**Traits**	**Types**	**Kernels**				
		**G**	**GK_A_**	**GK_D_**	**GK_ALL_**	**ALL**
KET	PCP	0.16	0.18	0.16	*0.19*	0.18
	EBV	0.85	0.86	0.84	*0.87*	0.86
DA	PCP	0.07	*0.08*	0.07	*0.08*	0.07
	EBV	0.59	*0.61*	0.53	0.59	0.60
RP	PCP	0.03	0.05	0.05	*0.06*	0.05
	EBV	0.65	*0.67*	0.60	0.66	0.65
LAME	PCP	0.07	*0.08*	0.04	0.07	0.05
	EBV	0.64	*0.66*	0.58	0.65	0.64
METR	PCP	0.05	*0.07*	0.04	0.05	0.05
	EBV	0.48	*0.52*	0.43	0.50	0.49
CM	PCP	0.07	*0.08*	0.05	0.07	0.07
	EBV	0.72	*0.74*	0.68	0.73	0.73

Pre-corrected phenotype (PCP) and estimated breeding value (EBV) were target phenotypes. Kernels were: additive genomic relationship kernel (**G**), Gaussian additive kernel (**GK**_**A**_), Gaussian dominance kernel (**GK**_**D**_), multiple kernel learning using Gaussian additive, Gaussian dominance, and Gaussian additive by dominance kernels (**GK**_**ALL**_), and fitting three parametric kernels (**G**, **D**, and **G**#**D**) simultaneously (**ALL**). The best prediction within trait and type of phenotype is italicized.

## Discussion

Recovering non-additive genetic variation in a validation dataset seems to be a challenge even when it is present, as appears to be the case for health traits in dairy cows. We observed that non-parametric kernels performed better irrespective of trait, but the predictive gain achieved over and above that from an additive genomic relationship kernel was small. Although mappings from genotypes to phenotypes may be captured more accurately with non-parametric kernels, recovering non-additive variance in CV remains an ongoing challenge in quantitative genetics. Arguably, use of environmental information, together with genomic data, may enhance predictive ability, especially of individual phenotypes such as PCP. This is an important topic for future research.

Quantifying non-additive genetic variances precisely requires setting up orthogonal additive, dominance, and additive by dominance epistasis kernels (Cockerham, 1954) and the assumption of no linkage and LE (Kempthorne, 1954). However, this is not feasible under linkage disequilibrium and selection. Therefore, the genetic variance decomposition obtained in this study via the three kernels should be taken as an approximation, because we cannot rule out the possibility that a single kernel captures multiple sources of genetic information.

In theory, non-additive genetic effects are of little relevance in genome-enabled selection, at least for most livestock species. Recently, Hansen (2013) argued from an evolutionary perspective that functional epistasis plays an important role in selection response, challenging the mainstream view of Hill et al. (2008). While additive genetic effects are expected to drive selection response based on Fisher's fundamental theorem under idealized conditions (Fisher, 1930; Crow, 2002), modeling non-additive effects explicitly might be required for proper estimation of breeding values and correct ranking of candidate parents for the next generation. Constructing four positive (semi) definite matrices (**G**, **D**, **GD**, and **I**) that are identifiable from one another seems indispensable to apportion genetic signals properly. An alternative approach is to construct **GD** = **X**_AD_ **X**′_AD_, where **X**_AD_ is the additive by dominance genotype matrix, but this requires intensive computation for *p* ≈ 50,000 (Xu, 2013).

It is important to note that Gaussian kernels pose a non-linear relationship between the kernel and SNP codes. If such a relationship holds, as may be the case for mean grain yield in wheat (e.g., Long et al., 2011a; Morota et al., 2013), an advantage should be detected. In this scenario, an upper bound for the squared predictive correlation obtained from non-parametric kernels would be broad sense heritability, as opposed to narrow sense heritability when an additive genomic relationship kernel is used (de los Campos et al., 2013b).

It is well known that there is no universal prediction machine that performs best on all cases and that the method of choice depends on the species, target trait, and possibly environmental circumstances. Nonetheless, this is a first report on the use of semi-parametric approaches for estimating marker-based non-additive genetic variances and predicting dairy cow health traits.

### Conflict of interest statement

The authors declare that the research was conducted in the absence of any commercial or financial relationships that could be construed as a potential conflict of interest.
